# Peritoneal dialysis catheter implantation: technical aspects, challenges, and current perspectives

**DOI:** 10.1590/21758239-JBN-2025-0267en

**Published:** 2026-02-23

**Authors:** Domingos Candiota Chula, Rodrigo Peixoto Campos, Ricardo Portiolli Franco, Henrique Carrascossi, Viviane Calice-Silva

**Affiliations:** 1 Universidade Federal do Paraná, Complexo Hospital de Clínicas, Curitiba, PR, Brazil.; 2 Fundação Pró-Renal Brasil, Curitiba, PR, Brazil.; 3 Universidade Federal do Paraná, Curitiba, PR, Brazil.; 4 Associação Renal Vida, Blumenau, SC, Brazil.; 5 Carrascossi Kidney Institute, Araraquara, SP, Brazil.; 6 Universidade de Araraquara, SP, Brazil.; 7 Fundação Pró-Rim, Joinville, SC, Brazil.; 8 Universidade da Região de Joinville, Joinville, SC, Brazil.

**Keywords:** Peritoneal Dialysis, Catheter, Techniques, Fluoroscopy, Procedure

## Abstract

The success of peritoneal dialysis (PD) depends on securing a functional, safe, and durable peritoneal access. Over recent decades, important advances in catheter design and insertion techniques have been consolidated, with the Tenckhoff catheter remaining the most widely used. Nevertheless, the prevalence of PD in Brazil remains limited, hindered by technical and logistical barriers to catheter placement, as well as by delays between indication and procedure. This review critically examines the main approaches to establishing PD access, with emphasis on technical aspects, clinical outcomes, and complications. Conventional surgery provides direct visualization of the peritoneal cavity in a simple and safe manner, whereas percutaneous methods, particularly those guided by ultrasonography and fluoroscopy, shorten hospitalization and broaden applicability when performed by nephrologists. Comparative evidence shows that percutaneous approaches achieve low rates of infectious and mechanical complications, with satisfactory catheter survival, while videolaparoscopic placement appears to yield superior results among the most frequently adopted techniques, despite greater technical and logistical complexity. Study heterogeneity limits definitive conclusions, underscoring the need for robust randomized clinical trials. Systematic use of imaging guidance may improve technical accuracy, while the active involvement of nephrologists in the procedure is crucial for reducing delays, optimizing outcomes, and expanding PD utilization. Standardization of practices and wider adoption of minimally invasive techniques represent promising avenues for strengthening PD as a therapeutic modality.

## Introduction

The effectiveness of peritoneal dialysis (PD) as a renal support therapy depends on establishing functional and durable peritoneal access. Since Ganter’s initial description in 1923, numerous catheter models and implantation techniques have been developed. The first devices used were adaptations of equipment originally intended for other purposes^1^. Over the following century, significant advances in materials and techniques made the method safer and more effective. In the 1960s, a study by Tenckhoff and Schechter resulted in the development of the most widely used catheter model to date, the Tenckhoff catheter^2^.

According to the 2024 Dialysis Census, more than 172,000 patients in Brazil are currently receiving dialysis therapy, of whom approximately 5.6% (around 9,600 patients) are on PD. Compared with previous surveys, the proportion of patients undergoing PD has shown a downward trend, with a slight recovery in the past year^3^. This scenario reinforces the importance of investigating factors contributing to the low prevalence of PD. Among the main factors are technical and logistical difficulties associated with peritoneal catheter implantation, as well as delays between patient admission and procedure performance. Nevertheless, complications associated with the catheter and its insertion are responsible for 5–10% of patients migrating to hemodialysis, often permanently^4,5^.

Various PD catheter implantation techniques have been described, differing according to catheter type and operator experience. The main approaches include conventional surgical implantation, percutaneous insertion with a trocar, peritoneoscopy, videolaparoscopy, and the Seldinger technique, with considerable heterogeneity in technical details used across different centers. Percutaneous puncture may be performed by the nephrologist at the bedside or in a procedure room, aiming to minimize the time between indication and access implantation. This approach can reduce hospitalization costs and positively impact the prevalence of PD as a dialysis modality^6,7^. In addition, the involvement of the nephrologist enables implants to be performed on an emergency basis, especially in situations where there is no time for elective scheduling^8^.

This review discusses the main technical aspects related to PD access, covering the evolution of the major implant methods, the associated challenges, and current prospects.

## General Care for Catheter Implantation

After a multidisciplinary assessment and decision regarding the implantation method, the first step in PD catheter placement is a preoperative evaluation, which should consider the patient’s physical characteristics, such as weight, height, and abdominal volume. Clothing habits should also be considered, as they influence technical aspects, including the choice of the catheter exit-site location. Adequate bowel preparation is also recommended, with laxatives and antipsychotics, as well as bladder emptying before the procedure, consistent with the International Society of Peritoneal Dialysis (ISPD) guidelines^9^. In the immediate preoperative period, there is evidence that antimicrobial prophylaxis significantly reduces the incidence of early catheter-related infections, and a single intravenous dose of a first- or second-generation cephalosporin is generally sufficient^10,11^.

During the procedure, several measures should be adopted to optimize catheter function and minimize complications. The ISPD recommends choosing an implantation side free of previous scars^9^, whereas European guidelines recommend the left side, avoiding the cecal region, although no comparative studies support this preference^12^. The catheter should ideally be inserted through the rectus abdominis muscle along the paramedian line, an approach that provides better fixation, a lower risk of dialysate leakage, and reduced hernia incidence. Traditionally, the internal cuff should be positioned inside the muscle or just above the internal fascia, avoiding entry into the abdominal cavity, which may promote adhesion formation^9,13^. However, a recent review proposes positioning both cuffs subcutaneously, without crossing the muscle layer, allowing early catheter use with low complication rates^14^.

Regarding the subcutaneous tunnel, some authors recommend a caudal and lateral orientation in relation to the inner cuff to reduce infectious complications. In a retrospective study of 1,930 patients, Golper et al.^15^ observed a 38% reduction in infectious complications with the caudal tunneling, while cranial orientation increased this rate by 50%^15^. In contrast, a prospective study by Crabtree and Burchette^16^ found no significant differences between caudal and lateral tunneling in 178 patients^16^. Current guidelines recommend that the tunnel shape should vary according to the catheter model. In straight catheters, for instance, the exit site may be positioned above the peritoneal entry point^9,17^. The external cuff should be positioned approximately 2 cm from the exit site, favoring epithelialization of the distal tunnel and reducing the risk of infection and accidental cuff extrusion^18^.

Postoperatively, hospitalization is typically not necessary, which contributes to reduced costs^19,20,21^. The guidelines recommend a “break-in” period of approximately 2 weeks before initiating dialysis. During this period, the exit site and surgical wound should remain covered with a dry dressing, avoiding local trauma^9,22^. Despite this recommendation, early catheter use has become common practice in several centers, with urgent-start PD representing a viable alternative^14,23^. During the break-in period, there are no formal recommendations for dialysate infusion, and saline flushing of the catheter should be avoided, as it may cause membrane irritation and catheter displacement^9,24,25^.

## Implantation Techniques

The development and dissemination of safe, effective, and low-cost techniques for PD catheter implantation that can be applied in clinical practice, particularly by nephrologists, are crucial for increasing access to this therapeutic modality. For better understanding and organization, the authors of this review have divided the implant modalities into three categories: trocar implantation (using the Tenckhoff trocar or more recent variations thereof), conventional surgical implantation (also referred to as minilaparotomy), and percutaneous implantation. Percutaneous implantation includes the Seldinger technique (and its variations) as well as fully percutaneous image-guided procedures, also called “minimally invasive” implants.

Percutaneous implantation with a trocar, originally described by Tenckhoff and Schechter in 1968^2^, is a simple technique with a low complication rate, although it is less commonly used today. Using only basic instruments, it remains useful in emergency settings or even in facilities with limited logistical resources, allowing early catheter placement with a minimized risk of dialysate leakage. In a retrospective study including 403 trocar implantations, Kang et al.^26^ reported early dysfunction in only 1% of catheters, bleeding in 1%, one episode of leakage, three exit-site infections, and 19 cases of peritonitis (4.7%). Late complications included extrusion of the external cuff (n = 3), leakage (n = 5), and catheter dysfunction (n = 9; 2.2%), with a survival rate of 96.5% at 1 year and 83.6% at 5 years^26^. In a comparative study involving 129 procedures, Chula et al.^27^ found no statistically significant differences between the trocar technique and the conventional surgical approach, both in terms of complications and catheter survival^27^.

The traditional surgical approach performed via minilaparotomy involves direct opening of the peritoneal cavity, allowing visualization of intra-abdominal structures and thereby reducing the risk of inadvertent visceral injury. This approach also allows omentectomy to be performed when indicated. In a study of 250 implants, Chow et al.^28^ reported catheter dysfunction in 2.8%, early peritonitis in 3.6%, and exit-site infection in 4.4%, with survival of 92.7% in the first year and 87.2% at the end of 2 years^28^.

Comparative studies of conventional surgical versus percutaneous techniques exhibit considerable heterogeneity. A meta-analysis encompassing 34 studies, most of which were observational, found that percutaneous implantation was associated with significantly lower rates of tunnel and exit-site infections, catheter migration, and the need for removal. However, no significant differences were observed in catheter survival^29^. When performed by nephrologists, PD catheter implantations are usually carried out percutaneously, using specific kits. When performed without imaging resources, percutaneous implantation does not allow direct or indirect real-time visualization of the peritoneal cavity, and puncture is performed blindly after skin incision and subcutaneous tissue dissection (Seldinger or modified Seldinger technique). Despite this limitation, it is a safe technique with good clinical results and the potential for immediate catheter use in emergency settings. In another meta-analysis including 1,626 patients that compared clinical outcomes between percutaneously and surgically implanted catheters, no significant difference in catheter survival at 1 year was observed; however, in general, the incidence of infectious and mechanical complications was significantly lower with percutaneous procedures^30^.

**Figure 1:**
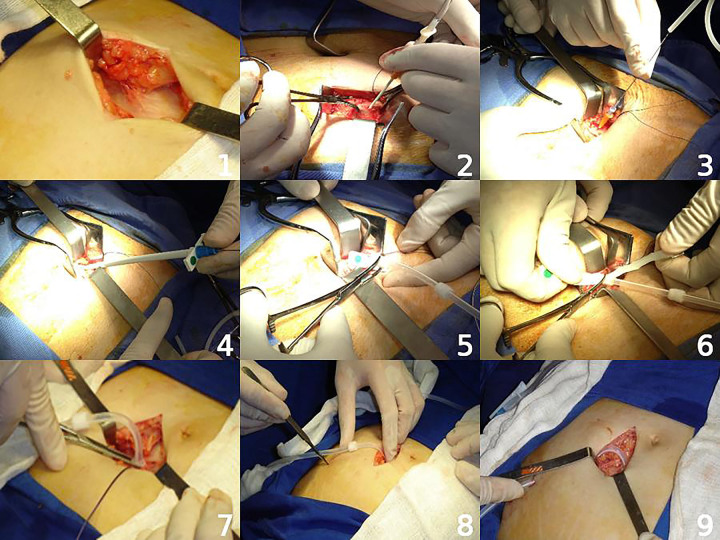
Main stages of peritoneal catheter implantation using the Seldinger technique.

The Seldinger technique, as described quite heterogeneously in the literature, shows wide variability in technical details. Owing to its broad applicability and its widespread use by nephrologists, the stages of catheter implantation using this method (as suggested by the authors and illustrated in Figure 1) are described below:

a) Asepsis and antisepsis under sterile draping;
b) Marking of the puncture site using the catheter as a reference (the catheter tip is positioned at the pubic symphysis and extended toward the paramedian line); the incision should be made at the point corresponding to the location of the internal cuff;
c) Local anesthesia of the skin and subcutaneous tissue;
d) Longitudinal or transverse incision, 2–5 cm long, followed by dissection of the subcutaneous tissue;
e) Visualization of the external aponeurosis of the rectus abdominis muscle, followed by puncture of the cavity using a kit needle or a peripheral intravenous catheter, at an angle of 45° or less relative to the abdominal wall;
f) Infusion of 500–1000 mL of 0.9% saline solution (optional), to promote ascites formation;
g) Insertion of the guidewire, with or without fluoroscopic assistance;
h) Passage of the dilator with sheath, followed by removal of the guidewire and dilator;
i) Insertion of the catheter through the sheath, which is subsequently torn and removed;
j) Fluoroscopic verification of positioning (optional); use of a rigid metal guidewire if necessary, followed by a drainage test;
k) Introduction of the internal cuff into the rectus abdominis muscle (optional);
l) Drainage test;
m) Cranial and lateral tunneling, followed by a repeat drainage test;
n) Suturing of the subcutaneous tissue with absorbable or semi-absorbable suture and skin closure with monofilament nylon;
o) Connection of the adapter and filling of the system with 5,000 IU of heparin in 9 mL of 0.9% saline solution (optional);
p) Dry compressive dressing.

(1) Dissection up to the external aponeurosis of the rectus abdominis muscle; (2) Puncture of the peritoneal cavity with an intravenous catheter; (3) Inserting the guidewire; (4) Inserting the introducer sheath with dilator; (5) Inserting the catheter through the sheath; (6) Sheath being torn and removed; (7) Placement of the internal cuff below the aponeurosis (optional); (8) Creation of the exit ostium; (9) Catheter already tunneled into the subcutaneous tissue.

In recent years, image-guided percutaneous techniques have been increasingly adopted, primarily to reduce mechanical complications. Ultrasound helps identify vessels, estimate cavity depth, detect adhesions, and facilitate anesthesia of the rectus abdominis muscle. Fluoroscopy provides real-time visualization for verifying proper catheter positioning. In a meta-analysis of 13 studies involving 2,681 patients, no significant difference was observed in the survival of surgically implanted catheters compared with image-guided catheters^31^. Similar results were later corroborated in a systematic review^32^. More recently, Swinnen et al.^33^ performed 61 implants with the aid of ultrasound and fluoroscopy. They reported technical success in 97% of cases, with leakage in 4.9% and the need for reintervention in only 3.2%^33^. In a Brazilian study, Chula et al.^34^ analyzed 74 image-guided implants (a technique referred to by the authors as “minimally invasive implantation”). They found lower mechanical and infectious complication rates compared with the trocar technique, with a oneyear survival rate of 89%^34^.

**Figure 2:**
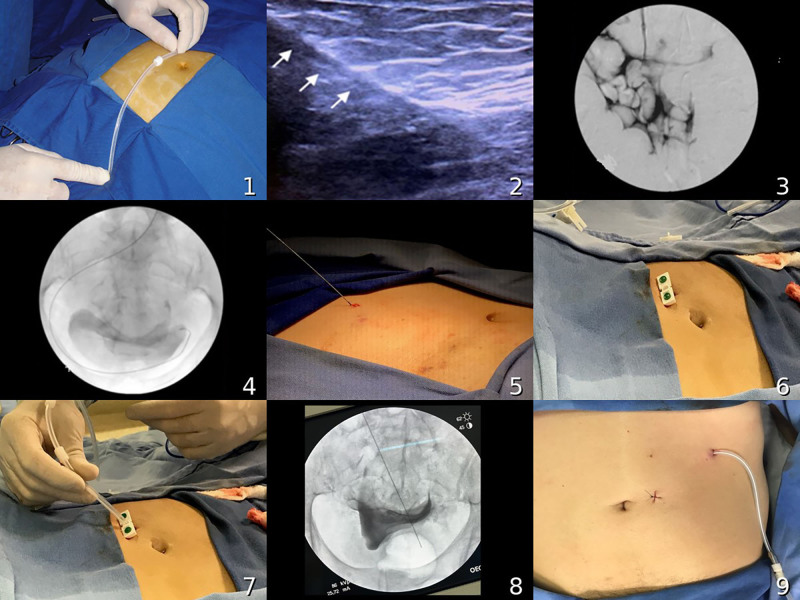
Main stages of image-guided (minimally invasive) peritoneal catheter implantation.

Image-guided percutaneous implantation combines low complication rates and excellent catheter survival with the convenience of being performed in an outpatient setting, making it a viable and effective alternative for nephrologists. The main steps (suggested by the authors and illustrated in Figure 2) for this procedure are described below:

a) Asepsis and antisepsis under sterile draping;
b) Marking of the puncture site, considering the position of the internal cuff (as previously described);
c) Local anesthesia of the skin and subcutaneous tissue;
d) Punctiform incision (<1 cm) and gentle blunt dissection of the subcutaneous tissue using hemostatic forceps;
e) Anesthesia of the rectus abdominis muscle, followed by peritoneal puncture with a 14F intravenous catheter under ultrasound guidance, using the smallest possible angle relative to the skin; removal of the needle, leaving only the flexible cannula within the abdominal cavity;
f) Peritoneography with iodinated contrast injection under fluoroscopy to confirm access to the cavity (contrast diffusion);
g) Infusion of 500–1000 mL of 0.9% saline solution under ultrasound visualization;
h) Insertion of the guidewire under fluoroscopy;
i) Insertion of the dilator with sheath, followed by removal of the guidewire and dilator;
j) Insertion of the catheter through the sheath, followed by sheath removal;
k) Fluoroscopic confirmation of position; a rigid metal guidewire may be used if necessary;
l) Deep positioning of the internal cuff within the subcutaneous tissue;
m) Drainage test;
n) Cranial and lateral tunneling, followed by a repeat drainage test;
o) Simple suturing with 3-0 monofilament nylon suture;
p) Connection of the adapter and filling of the system with 5,000 IU of heparin in 9 mL of 0.9% saline solution (optional);
q) Dry compressive dressing.

(1) Measurement to determine the incision site; (2) Ultrasound-guided puncture of the peritoneal cavity; (3) Peritoneography; (4) Passage of the guidewire under fluoroscopic guidance; (5) Guidewire introduced; (6) Sheath introduced; (7) Insertion of the catheter through the sheath; (8) Checking the correct positioning of the catheter tip in the minor pelvis; (9) Final result.

The use of peritoneoscopy or laparoscopy allows direct visualization of the abdominal cavity, thereby reducing complication rates, especially when performed by experienced professionals. In addition, the laparoscopic approach enables the use of advanced surgical techniques, such as prophylactic omentopexy and adhesiolysis. However, these methods are generally reserved for patients considered suitable for general anesthesia and willing to wait for an available operating room. A meta-analysis conducted by Shrestha et al.^35^ showed a significant reduction in flow obstruction and catheter tip migration when advanced laparoscopic techniques were used^35^. More recently, Briggs et al.^36^, in a new meta-analysis, demonstrated that laparoscopic insertion of PD catheters, compared with the conventional surgical approach, resulted in little or no difference in the risks of peritonitis, catheter removal, and dialysate leakage, but did reduce the risk of bleeding and catheter tip migration^36^. In a recent retrospective study, Zheng et al.^37^ compared the outcomes of implants performed using advanced laparoscopic techniques and image-guided percutaneous approaches (ultrasound- and fluoroscopy-assisted). The authors observed greater catheter survival and a lower incidence of minor bleeding with laparoscopic procedures. However, the median time between the indication and the procedure was significantly longer for laparoscopic techniques than for percutaneous techniques (33 versus 12 days; p < 0.001)^37^.

## Complications

Complications related to PD catheter implantation and maintenance can be divided into two main categories: mechanical (or non-infectious) and infectious. Among the mechanical complications, perforation of hollow viscera, although rare, is the most feared event during catheter implantation. When it occurs, it can typically be identified and managed relatively easily in procedures that allow direct visualization. Management may be conservative, with antimicrobial therapy, or may require surgical intervention, depending on severity^38,39^. Another complication observed in the first few days after implantation is the presence of a bloody dialysate (hemoperitoneum), with severe bleeding being less frequent and generally related to vascular lesions, such as injury to the inferior epigastric artery. Mital et al.^40^ reported a 2% rate of major bleeding, associated with factors such as anticoagulant use, uremia, thrombocytopenia, or acetylsalicylic acid^40^. Intraperitoneal bleeding is an independent risk factor for adhesion formation and catheter failure^41^. In this sense, the use of color Doppler ultrasound during abdominal wall puncture helps to locate the inferior epigastric artery or other larger vessels, thereby minimizing the risk of inadvertent puncture and consequent bleeding.

Catheter dysfunction may manifest as low flow in the infusion or drainage of dialysate and is often associated with migration of the intraperitoneal tip, which occurs in 15% to 20% of cases, although it does not always result in malfunction^42,43^. Dysfunctions may be related to the involvement of the catheter by the omentum, and procedures such as omentopexy are effective in preventing these complications^44^. When displacement occurs, maneuvers such as the use of flexible metal guides or videolaparoscopy may help reposition the catheter^45,46^. Similarly, among the mechanical complications, dialysate leakage, whether early or late, may manifest as fluid leakage from the exit site or as subcutaneous collections, affecting up to 5% of patients. The diagnosis is usually made on inspection or by CT scan with intraperitoneal contrast; its treatment includes peritoneal rest and, in some cases, surgical intervention^42,47^. Extrusion of the external cuff, in contrast, typically results from flaws in the implantation technique, particularly when it is positioned too close to the exit site. In the absence of internal cuff infection or peritonitis, it is possible to maintain the catheter with careful shaving of the externalized cuff^48^.

Among infectious complications, peritonitis stands out as a serious event that can occur at any time after catheter implantation and is strongly associated with dialysis technique failure. The ISPD recommends an incidence of less than 0.4 episodes per patient-year, and recurrent or refractory cases may require definitive catheter removal^11,49,50^. Exit-site infection, characterized by purulent secretion and local hyperemia, should be treated with appropriate antimicrobials. Its incidence ranges from 0.05 to 1.02 episodes per patient-year and is strongly associated with peritonitis. Tunnel infection, usually associated with infection of the exit site, can be diagnosed on physical examination or by ultrasound. Although it rarely occurs in isolation, it may cause recurrent peritonitis and requires specific antimicrobial treatment^51^.

In addition to these complications, there are reports of rare events, such as erosion of intestinal loops, catheter fragmentation, and allergic reactions to silicone, which, although uncommon, should be considered in the clinical follow-up of patients^52,53,54^.

Considering the heterogeneity of implantation techniques described in the literature, comparing complication rates related to the implantation and maintenance of PD catheters is a challenging task. Table 1 presents the incidence of complications, according to different insertion techniques, as reported in studies published over the past 15 years. To highlight the main mechanical and infectious complications, it was necessary, in some cases, to group certain outcomes.

**Table 1:** Complications related to peritoneal dialysis catheter implantation and maintenance, according to the implantation technique used

First author (year)	Technique	N	Catheter dislodgement	Drainage problems	Leakage	Bleeding	Early peritonitis	Tunnel infection	Exit site infection	Other complications	Catheter survival at 1 year
Jwo et al. (2010)^55^	Laparoscopy	37	10.8	NS	21.6	21	NS	NS	16.2	5.4	82
Kou et al. (2023)^56^	Laparoscopy	440	NS	5.9	NS	NS	NS	NS	NS	NS	NS
Iorga et al. (2024)^57^	Laparoscopy	126	4.3	NS	21.7	4.3	3.1	NS	30.4	NS	NS
Ìslam et al. (2023)^58^	Laparoscopy	24	25	NS	33.3	25	NS	NS	8.3	4.3	NS
Jwo et al. (2010)^55^	Laparotomy	40	17.5	NS	17.5	7.5	NS	NS	12.5	2.5	86
Chula et al. (2014)^27^	Laparotomy	42	NS	9.5	0	9.5	2.3	2.3	NS	0	76
Al-Hwiesh AK (2014)^59^	Laparotomy	40	7.5	12.5	2.5	0	0	0	17.5	2.5	87.5
Zhang et al. (2020)^60^	Laparotomy	114	0	0.9	NS	1.8	NS	NS	2.6	1.8	NS
Dogra et al. (2018)^61^	Laparotomy	105	3.8	0.9	0	7.6	NS	0	2.8	0.9	71.8
Aksu et al. (2007)^62^	Seldinger	108	9.2	5.5	NS	NS	NS	1.8	NS	5.5	92.4
Zou et al. (2021)^63^	Seldinger	94	22.5	15.5	9	2.2	NS	NS	NS	0	NS
Chula et al. (2023)^34^	Seldinger	73	6.8	0	0	3.9	1.3	0	3.9	2.6	95
Ìslam et al. (2023)^58^	Seldinger	36	19.4	NS	11.1	19.4	NS	NS	0	5.6	NS
Huang et al. (2022)^64^	Seldinger	34	0	2.9	2.9	17.6	0	0	0	2.9	NS
Dias et al. (2017)^65^	Seldinger	51	15.6	NS	7.8	NS	NS	0	17	3.8	NS
Chula et al. (2014)^27^	Trocarte	79	NS	12.6	0	6.3	2.5	2.5	NS	1.2	82
Lee et al. (2015)^66^	Trocarte	148	1.2	9.8	16.3	13.3	NS	NS	16.7	0	NS
Peng et al. (2019)^67^	Trocarte	84	NS	10.7	1.2	1.2	0	0	0	0	NS
Li et al. (2020)^68^	Trocarte	103	1	4.7	0	0	0	0	NS	3.8	92.2
Zhang et al. (2022)^69^	Trocarte	280	2.5	5.3	1	0	1.5	0	1	2.5	NS
Zhang et al. (2020)^70^	Trocarte	126	1.6	2.4	NS	1.6	NS	NS	0.8	1.6	NS

Abbreviation – NS: not specified.

In conclusion, despite significant advances in PD catheter implantation techniques, important challenges remain. The authors note the need for well-designed randomized clinical trials that robustly compare the main techniques currently in use. At the same time, the systematic use of imaging technologies and resources is essential to improve technical precision and reduce complications. Nevertheless, the active involvement of nephrologists in these procedures remains essential to optimize clinical outcomes and encourage the expansion of PD as a therapeutic modality.

## Data Availability

This article is a literature review and is based exclusively on secondary data obtained from previously published studies, as well as the pro­fessional experience of the authors. Since no primary data were generated and no novel datasets were compiled for this work, there are no additional data files to be made available. All information analyzed in this review can be accessed through the sources referenced in the manuscript.
