# Supplementary material for: Peritoneal dialysis catheter implantation: technical aspects, challenges, and current perspectives
Source: J Bras Nefrol. 2026 Feb 23;48(2):e20250267. doi: 10.1590/21758239-JBN-2025-0267en (PMC12928706; doi:10.1590/21758239-JBN-2025-0267en)
Supplement: Translation

# Translation: Implante do cateter para diálise peritoneal: aspectos técnicos, desafios e perspectivas atuais

**DOI:** 10.1590/21758239-JBN-2025-0267pt

**Authors:** Domingos Candiota Chula, Rodrigo Peixoto Campos, Ricardo Portiolli Franco, Henrique Carrascossi, Viviane Calice-Silva

**Affiliations:** 1 Universidade Federal do Paraná, Complexo Hospital de Clínicas, Curitiba, PR, Brasil.; 2 Fundação Pró-Renal Brasil, Curitiba, PR, Brasil.; 3 Universidade Federal do Paraná, Curitiba, PR, Brasil.; 4 Associação Renal Vida, Blumenau, SC, Brasil.; 5 Carrascossi Kidney Institute, Araraquara, SP, Brasil.; 6 Universidade de Araraquara, SP, Brasil.; 7 Fundação Pró-Rim, Joinville, SC, Brasil.; 8 Universidade da Região de Joinville, Joinville, SC, Brasil.

**Keywords:** Diálise Peritoneal, Cateteres, Técnicas, Fluoroscopia, Procedimento

## Abstract

O êxito da diálise peritoneal (DP) depende da obtenção de um acesso peritoneal funcional, seguro e duradouro. Nas últimas décadas, avanços no desenho dos cateteres e nas técnicas de inserção foram consolidados, destacando-se o cateter de Tenckhoff como o mais utilizado. Apesar disso, a prevalência da DP no Brasil permanece reduzida, condicionada por limitações técnicas e logísticas no implante do cateter e pelo intervalo entre a indicação do método e a realização do procedimento. Esta revisão analisa criticamente as principais modalidades de implantação do cateter para DP, enfatizando aspectos técnicos, desfechos clínicos e complicações. A cirurgia convencional possibilita visualização direta da cavidade de forma simples e segura, enquanto as técnicas percutâneas, sobretudo as guiadas por ultrassonografia e fluoroscopia, reduzem o tempo de hospitalização e ampliam a aplicabilidade do procedimento pelo nefrologista. Evidências comparativas demonstram que as abordagens percutâneas apresentam baixas taxas de complicações infecciosas e mecânicas, com boa sobrevida do cateter, enquanto os implantes videolaparoscópicos parecem alcançar melhores resultados entre as modalidades mais utilizadas, embora com maior complexidade técnica e logística. A heterogeneidade dos estudos limita conclusões definitivas, reforçando a necessidade de ensaios clínicos randomizados robustos. Uma utilização sistemática de métodos de imagem pode ampliar a acurácia técnica, enquanto a participação ativa do nefrologista no implante é determinante para reduzir atrasos, otimizar desfechos clínicos e expandir o uso da DP. A padronização dos procedimentos e a difusão de técnicas minimamente invasivas configuram perspectivas promissoras para o fortalecimento da DP como modalidade terapêutica.

## Introdução

O sucesso da diálise peritoneal (DP) como terapia de suporte renal depende de um acesso peritoneal funcional e duradouro. Desde a descrição inicial por Ganter, em 1923, diversos modelos de cateteres e técnicas de implantação foram desenvolvidos. Os primeiros dispositivos utilizados eram adaptações de equipamentos destinados a outras finalidades^1^. Ao longo dos cem anos seguintes, ocorreram avanços significativos em materiais e técnicas, tornando o método mais seguro e eficaz. Destacam-se, na década de 1960, os estudos de Tenckhoff e Schechter, que resultaram no modelo de cateter mais amplamente utilizado até hoje, o “cateter de Tenckhoff”^2^.

Segundo o Censo de Diálise de 2024, mais de 172.000 pacientes estão em terapia dialítica no Brasil, sendo aproximadamente 5,6% (cerca de 9.600 pacientes) em DP. Em comparação a levantamentos anteriores, observa-se uma tendência de queda na proporção de pacientes em DP, com leve recuperação no último ano^3^. Esse cenário reforça a importância de investigar os fatores que contribuem para a baixa prevalência da modalidade. Entre os principais fatores estão dificuldades técnicas e logísticas relacionadas à implantação do cateter peritoneal, além do tempo de espera entre a admissão do paciente e a realização do procedimento. Não obstante, complicações associadas ao cateter e à sua inserção são responsáveis pela migração de 5% a 10% dos pacientes para a hemodiálise, muitas vezes de forma definitiva^4,5^.

Diversas técnicas para a implantação do cateter de DP estão descritas, variando conforme o tipo de cateter e a experiência do operador. Entre as principais estão o implante cirúrgico convencional, a inserção percutânea com trocarte, a peritonioscopia, a videolaparoscopia e a técnica de Seldinger, havendo grande heterogeneidade nos detalhes técnicos empregados em cada uma delas em diferentes centros. A punção percutânea pode ser realizada pelo próprio nefrologista, em sala de procedimento ou à beira do leito, com o objetivo de reduzir o tempo entre a indicação e a implantação do acesso; essa abordagem pode diminuir os custos de internação e impactar positivamente na prevalência da DP como modalidade dialítica^6,7^. Além disso, o envolvimento do nefrologista possibilita a realização de implantes em caráter emergencial, especialmente em situações nas quais não há tempo hábil para programação eletiva^8^.

A presente revisão tem como objetivo discutir os principais aspectos técnicos relacionados ao acesso para DP, abordando a evolução das principais modalidades de implante, os desafios encontrados e as perspectivas atuais.

## Cuidados Gerais para a Implantação do Cateter

Após avaliação multidisciplinar e decisão pelo método, o primeiro passo para a implantação do cateter de DP é a avaliação pré-operatória, que deve considerar características físicas como peso, altura e volume abdominal. Os hábitos de vestuário também devem ser considerados, pois influenciam aspectos técnicos, como a escolha do sítio de saída do cateter. Recomenda-se ainda um preparo intestinal adequado, com laxativos e antifiséticos, bem como o esvaziamento vesical antes do procedimento, conforme diretrizes da *International Society for Peritoneal Dialysis* (ISPD)^9^. No pré-operatório imediato, há evidências de que a profilaxia antimicrobiana reduz significativamente a incidência de infecções precoces relacionadas ao cateter, sendo geralmente suficiente a administração de uma dose única endovenosa de cefalosporina de primeira ou segunda geração^10,11^.

Durante o procedimento, diversas medidas devem ser adotadas para otimizar o funcionamento do cateter e minimizar complicações. A ISPD recomenda a escolha do lado de implantação livre de cicatrizes prévias^9^, enquanto diretrizes europeias sugerem o lado esquerdo, evitando a região do ceco, embora não existam estudos comparativos que sustentem essa preferência^12^. A introdução do cateter deve ocorrer, idealmente, através do músculo reto abdominal, na linha paramediana, abordagem que proporciona melhor fixação, menor risco de extravasamento do dialisato e menor incidência de hérnias. Tradicionalmente, o manguito interno deve ser posicionado dentro do músculo ou acima da fáscia interna, evitando sua introdução na cavidade abdominal, o que pode levar à formação de aderências^9,13^. No entanto, uma revisão recente propõe o posicionamento de ambos os manguitos no tecido subcutâneo, sem atravessar a camada muscular, possibilitando, inclusive, o uso precoce do cateter com baixas taxas de complicações^14^.

Quanto ao túnel subcutâneo, alguns autores recomendam sua orientação caudal e lateral em relação ao manguito interno, buscando reduzir complicações infecciosas. Em um estudo retrospectivo com 1.930 pacientes, Golper et al.^15^ observaram redução de 38% nas complicações infecciosas com túnel voltado para caudal, enquanto a orientação cranial aumentou essa taxa em 50%^15^. Em outro estudo prospectivo, Crabtree e Burchette^16^ não encontraram diferenças significativas entre túnel caudal e lateral em 178 pacientes^16^. As diretrizes atuais recomendam que a conformação do túnel varie conforme o modelo do cateter. Em cateteres retos, por exemplo, o sítio de saída pode ser posicionado acima do ponto de entrada na cavidade peritoneal^9,17^. Também se recomenda que o manguito externo fique a cerca de 2 cm do orifício de saída, favorecendo a epitelização do túnel distal e reduzindo o risco de infecção e extrusão acidental do manguito^18^.

No pós-operatório, geralmente não é necessária internação hospitalar, o que contribui para a redução de custos^19,20,21^. As diretrizes sugerem um período de espera (*break-in*) de aproximadamente duas semanas antes do início da diálise, durante o qual o orifício de saída e a ferida operatória devem permanecer cobertos com curativo seco, evitando traumas locais^9,22^. Apesar disso, o uso precoce do cateter tem se tornado prática comum em diversos centros, sendo a DP de início urgente uma alternativa viável^14,23^. Durante o *break-in*, não há recomendações formais para a infusão de dialisato, e a lavagem peritoneal com solução salina deve ser evitada, pois pode causar irritação da membrana e deslocamento do cateter^9,24,25^.

## Técnicas de Implante

O desenvolvimento e a disseminação de técnicas de implante de cateteres para DP que sejam seguras, eficazes, de baixo custo e que possam ser aplicadas na prática clínica, particularmente por nefrologistas, são cruciais para ampliar o acesso a essa modalidade terapêutica. Para melhor compreensão e organização, os autores desta revisão dividiram as modalidades de implante em três categorias: implantes por trocarte (utilizando o trocarte de Tenckhoff ou outras variações mais atuais desse dispositivo), implantes cirúrgicos convencionais (também chamados de minilaparotomia) e implantes percutâneos. Entre os implantes percutâneos estão o implante por Seldinger (e suas variações) e os implantes totalmente percutâneos guiados por imagem, aqui também denominados implantes “minimamente invasivos”.

O implante percutâneo com trocarte, descrito originalmente por Tenckhoff e Schechter em 1968^2^, é uma técnica simples e com baixa taxa de complicações, embora seja pouco utilizada atualmente. Utilizando apenas instrumental básico, pode ser útil em situações emergenciais ou em locais com poucos recursos logísticos, permitindo o uso precoce do cateter com risco minimizado de extravasamento de dialisato. Em um estudo retrospectivo que incluiu 403 implantes realizados por trocarte, Kang et al.^26^ observaram disfunção precoce em somente 1% dos cateteres, sangramentos também em 1%, um único episódio de extravasamento, três infecções do sítio de saída e 19 casos de peritonite (4,7%). As complicações tardias incluíram extrusão do manguito externo (n = 3), extravasamento (n = 5) e disfunção do cateter (n = 9; 2,2%), com uma taxa de sobrevida de 96,5% em um ano e 83,6% em cinco anos^26^. Em outro estudo comparativo envolvendo 129 procedimentos, Chula et al.^27^ não observaram diferenças estatisticamente significativas entre a técnica por trocarte e a abordagem cirúrgica convencional, tanto no que diz respeito às complicações quanto à sobrevida dos cateteres^27^.

O implante cirúrgico tradicional, por meio de minilaparotomia, envolve a abertura direta da cavidade peritoneal, permitindo a visualização das estruturas intra-abdominais, o que reduz o risco de lesões viscerais inadvertidas. Essa abordagem também possibilita a realização de omentectomia, quando indicada. Em um estudo com 250 implantes, Chow et al.^28^ relataram disfunção do cateter em 2,8%, peritonite precoce em 3,6% e infecção do sítio de saída em 4,4%, com sobrevida de 92,7% no primeiro ano e 87,2% ao final de dois anos^28^.

Os estudos que comparam a técnica cirúrgica convencional com os procedimentos percutâneos são bastante heterogêneos. Em uma metanálise que incluiu 34 estudos, em sua maioria observacionais, o implante percutâneo esteve associado a taxas significativamente menores de infecção do túnel e do sítio de saída, migração do cateter e necessidade de remoção. No entanto, não foram encontradas diferenças significativas na sobrevida dos cateteres^29^. Quando realizados por nefrologistas, os implantes de cateter para DP são, habitualmente, feitos por via percutânea, com uso de kits específicos. Quando realizado sem o emprego de recursos de imagem, o implante percutâneo não permite visualização direta ou indireta da cavidade peritoneal em tempo real, sendo a punção realizada de forma cega após incisão da pele e dissecção do tecido subcutâneo (técnica de Seldinger ou Seldinger modificada). Apesar dessa limitação, trata-se de uma técnica segura, com bons resultados clínicos e possibilidade de uso imediato do cateter em situações de urgência. Em outra metanálise, que incluiu 1.626 pacientes e comparou os desfechos clínicos entre cateteres implantados por técnica percutânea e por via cirúrgica, não houve diferença significativa na sobrevida dos cateteres em um ano; contudo, de modo geral, a incidência de complicações infecciosas e mecânicas foi significativamente menor nos procedimentos percutâneos^30^.

A descrição da técnica de Seldinger é bastante heterogênea na literatura, com inúmeras variações em seus detalhes técnicos. Devido à sua alta aplicabilidade, amplamente utilizada por nefrologistas, descrevemos abaixo as etapas (sugeridas pelos autores e ilustradas na Figura 1) do implante de cateter por este método:

a) Assepsia e antissepsia, com colocação de campos estéreis;
b) Marcação do ponto de punção, utilizando o cateter como medida (posiciona-se a ponta do cateter na sínfise púbica, esticando-o em direção à linha paramediana); no ponto correspondente à localização do manguito interno, deve ser realizada a incisão;
c) Anestesia local da pele e do subcutâneo;
d) ncisão longitudinal ou transversal, com 2 a 5 cm de extensão, seguida de dissecção do subcutâneo;
e) Visualização da aponeurose externa do músculo reto abdominal, seguida da punção da cavidade com agulha do kit ou cateter intravenoso periférico, em uma angulação em relação à parede abdominal de 45° ou inferior;
f) Infusão de 500–1000 mL de SF 0,9% para formação de ascite (opcional);
g) Passagem do fio-guia, com ou sem uso da fluoroscopia;
h) Passagem do dilatador com bainha, seguida da retirada do guia e dilatador;
i) Inserção do cateter pela bainha, a qual é então rasgada e retirada;
j) Fluoroscopia para verificar o posicionamento (opcional); uso de fio-guia metálico rígido, se necessário, seguido de teste de drenagem;
k) Introdução do manguito interno no músculo reto abdominal (opcional);
l) Teste de drenagem;
m) Tunelização cranial e lateral, com novo teste de drenagem;
n) Sutura do subcutâneo com fio absorvível ou semiabsorvível e da pele com fio mononylon;
o) Conexão do adaptador e preenchimento do sistema com 5.000 UI de heparina em 9 mL de SF 0,9% (opcional);
p) Curativo compressivo seco.

**Figura 1:** Principais etapas do implante do cateter peritoneal pela técnica de Seldinger.

(1) Dissecção até a aponeurose externa no músculo reto abdominal; (2) Punção da cavidade peritoneal com cateter intravenoso; (3) Passagem do fio guia; (4) Passagem da bainha introdutora com dilatador; (5) Passagem do cateter através da bainha; (6) Bainha sendo rasgada e retirada; (7) Introdução do manguito interno abaixo da aponeurose (opcional); (8) Criação do óstio de saída; (9) Cateter já tunelizado em subcutâneo.

Mais recentemente, técnicas percutâneas guiadas por imagem têm sido adotadas com o objetivo de reduzir, especialmente, as complicações mecânicas. A ultrassonografia auxilia na identificação de vasos, na estimativa da profundidade da cavidade e na detecção de aderências, além de facilitar a anestesia do músculo reto abdominal. A fluoroscopia contribui para a verificação, em tempo real, do posicionamento adequado do cateter. Em metanálise com 13 estudos e 2.681 pacientes, não houve diferença significativa na sobrevida dos cateteres implantados cirurgicamente em comparação aos guiados por imagem^31^. Resultados semelhantes foram corroborados, posteriormente, em uma revisão sistemática^32^. Mais recentemente, Swinnen et al.^33^, em 61 implantes realizados com auxílio de ultrassonografia e fluoroscopia, relataram sucesso técnico em 97% dos casos, com extravasamento em 4,9% e necessidade de reintervenção em apenas 3,2%^33^. Em um estudo brasileiro, Chula et al.^34^ analisaram 74 implantes guiados por imagem (técnica batizada pelos autores como “implante minimamente invasivo”) e observaram menor taxa de complicações mecânicas e infecciosas em comparação à técnica com trocarte, com sobrevida de 89% em um ano^34^.

O implante percutâneo guiado por imagem associa baixa taxa de complicações à excelente sobrevida do cateter, além de possibilitar sua realização em regime ambulatorial, o que torna essa técnica uma alternativa viável e eficaz para nefrologistas. Dessa forma, descrevemos abaixo as principais etapas (sugeridas pelos autores e ilustradas na Figura 2) para este procedimento:

a) Assepsia e antissepsia, com colocação de campos estéreis;
b) Marcação do ponto de punção, considerando a posição do manguito interno (conforme descrito acima);
c) Anestesia local da pele e do subcutâneo;
d) Incisão puntiforme (<1 cm) e discreto divulsionamento do subcutâneo com pinça hemostática;
e) Anestesia do músculo reto abdominal e punção peritoneal com cateter intravenoso 14F, guiada por ultrassonografia, empregando a menor angulação possível em relação à pele, com retirada da agulha e manutenção somente da cânula flexível na cavidade abdominal;
f) Peritoniografia com injeção de contraste iodado, sob fluoroscopia, para confirmação do acesso à cavidade (espalhamento do contraste);
g) Infusão de 500–1000 mL de SF 0,9% sob visualização ultrassonográfica;
h) Passagem do fio-guia sob fluoroscopia;
i) Passagem do dilatador com bainha, seguida da retirada do guia e dilatador;
j) Inserção do cateter pela bainha e posterior retirada da mesma;
k) Fluoroscopia para verificação do posicionamento; uso de fio metálico rígido, se necessário;
l) Posicionamento profundo do manguito interno no subcutâneo;
m) Teste de drenagem;
n) Tunelização cranial e lateral, com novo teste de drenagem;
o) Sutura simples com fio mononylon 3-0;
p) Conexão do adaptador e preenchimento do sistema com 5.000 UI de heparina em 9ml de SF 0,9% (opcional);
q) Curativo compressivo seco.

**Figura 2:** Principais etapas do implante do cateter peritoneal guiado por imagem (minimamente invasivo).

(1) Medida para localização do ponto de incisão; (2) Punção da cavidade peritoneal guiada por ultrassom; (3) Peritoniografia; (4) Passagem do fio guia sob flouroscopia; (5) Fio guia introduzido; (6) Bainha introduzida; (7) Introdução do cateter através da bainha; (8) Checagem do correto posicionamento da ponta do cateter na pelve menor; (9) Resultado final.

A utilização da peritonioscopia ou da laparoscopia permite a visualização direta da cavidade abdominal, o que contribui para a redução de complicações, especialmente quando realizadas por profissionais experientes. Além disso, a abordagem videolaparoscópica possibilita a execução de técnicas cirúrgicas avançadas, como a omentopexia profilática e a adesiólise. No entanto, essa abordagem é geralmente reservada para pacientes considerados aptos à anestesia geral e com disponibilidade para aguardar vaga em centro cirúrgico. A metanálise conduzida por Shrestha et al.^35^ evidenciou redução significativa na obstrução do fluxo e na migração da ponta do cateter quando técnicas laparoscópicas avançadas foram utilizadas^35^. Mais recentemente, Briggs et al.^36^, em nova metanálise, demonstraram que a inserção laparoscópica de cateteres para DP, em comparação à técnica cirúrgica convencional, apresenta pouca ou nenhuma diferença nos riscos de peritonite, remoção do cateter e extravazamento de dialisato, mas pode reduzir o risco de hemorragias e da migração da ponta do cateter^36^. Em estudo retro­spectivo recente, Zheng et al.^37^ compararam os desfechos de implantes realizados por videolaparoscopia avançada com aqueles obtidos por técnicas percutâneas guiadas por imagem (ultrassonografia e fluoroscopia). Os autores observaram maior sobrevida dos cateteres e menor incidência de sangramentos leves nos procedimentos laparoscópicos. Contudo, o tempo mediano entre a indicação e a realização do procedimento foi significativamente maior nas técnicas laparoscópicas, em comparação às técnicas percutâneas (33 versus 12 dias; p < 0,001)^37^.

## Complicações

As complicações relacionadas ao implante e à manutenção do cateter de DP podem ser divididas em duas categorias principais: mecânicas (ou não infecciosas) e infecciosas. Entre as complicações mecânicas, a perfuração de vísceras ocas, embora rara, é a mais temida durante o implante do cateter. Quando ocorre, costuma ser identificada e tratada com relativa facilidade em procedimentos com visualização direta, podendo o manejo ser conservador, com antimicrobianos, ou exigir intervenção cirúrgica, a depender da gravidade^38,39^. Outra complicação observada nos primeiros dias após o implante é a presença de dialisato com sangue (hemoperitônio), sendo os sangramentos volumosos menos frequentes e geralmente relacionados a lesões vasculares, como as da artéria epigástrica inferior. Mital et al.^40^ relataram uma taxa de 2% de sangramentos importantes, associados a fatores como uso de anticoagulantes, uremia, trombocitopenia ou ácido acetilsalicílico^40^. O sangramento intraperitoneal constitui um fator de risco independente para a formação de aderências e para a falência do cateter^41^. Nesse sentido, o uso da ultrassonografia com *Color Doppler*, durante a punção da parede abdominal, ajuda a localizar a artéria epigástrica inferior ou outros vasos de maior calibre, minimizando o risco de uma punção inadvertida e do consequente sangramento.

A disfunção do cateter pode se manifestar como baixo fluxo na infusão ou na drenagem do dialisato e está frequentemente associada à migração da extremidade intraperitoneal, que ocorre em 15% a 20% dos casos, embora nem sempre resulte em mau funcionamento^42,43^. As disfunções podem estar relacionadas ao envolvimento do cateter pelo omento, e procedimentos como a omentopexia têm se mostrado eficazes na prevenção dessas intercorrências^44^. Quando ocorre o deslocamento, manobras como o uso de guias metálicos flexíveis ou a videolaparoscopia podem auxiliar no reposicionamento do cateter^45,46^. Ainda entre as complicações mecânicas, o extravasamento de dialisato, precoce ou tardio, pode se manifestar como saída de líquido pelo orifício de saída ou pela formação de coleções subcutâneas, afetando até 5% dos pacientes. O diagnóstico é comumente realizado já na inspeção ou por tomografia computadorizada com contraste intraperitoneal; seu tratamento inclui repouso peritoneal e, em alguns casos, intervenção cirúrgica^42,47^. A extrusão do manguito externo, por sua vez, decorre geralmente de falhas na técnica de implante, especialmente quando este é posicionado muito próximo ao orifício de saída. Na ausência de infecção do manguito interno ou peritonite, é possível manter o cateter com raspagem cuidadosa do manguito exteriorizado^48^.

Entre as complicações infecciosas, destaca-se a peritonite, intercorrência grave que pode ocorrer a qualquer momento após o implante do cateter e está fortemente associada à falência da técnica dialítica. A ISPD recomenda uma incidência inferior a 0,4 episódios por paciente-ano, sendo que casos recorrentes ou refratários podem demandar a retirada definitiva do cateter^11,49,50^. A infecção do sítio de saída, caracterizada por secreção purulenta e hiperemia local, deve ser tratada com antimicrobianos adequados. Sua incidência varia entre 0,05 e 1,02 episódios por paciente-ano e está fortemente associada à ocorrência de peritonite. A infecção do túnel, geralmente associada à infecção do sítio de saída, pode ser diagnosticada ao exame físico ou por ultrassonografia; embora raramente ocorra de forma isolada, pode causar peritonites recorrentes e exige tratamento antimicrobiano específico^51^.

Além dessas complicações, há relatos de intercorrências raras, como erosão de alças intestinais, fragmentação do cateter e reações alérgicas ao silicone, que, embora incomuns, devem ser consideradas no acompanhamento clínico dos pacientes^52,53,54^.

Considerando a heterogeneidade das técnicas de implantação descritas na literatura, a comparação das taxas de complicações relacionadas ao implante e à manutenção dos cateteres de DP torna-se uma tarefa desafiadora. A Tabela 1 apresenta as incidências de complicações, de acordo com diferentes técnicas de inserção, reportadas em estudos publicados nos últimos 15 anos. Com o objetivo de evidenciar as principais complicações mecânicas e infecciosas, foi necessário, em alguns casos, agrupar determinados desfechos.

**Tabela 1:** Complicações relacionadas ao implante e à manutenção do cateter para diálise peritoneal, de acordo com a técnica de implantação utilizada

Primeiro Autor (ano)	Técnica	N	Deslocamento do cateter	Problemas de drenagem	Extravasamento	Sangramento	Peritonite precoce	Infecção de túnel	Infecção de sítio de saída	Outras complicações	Sobrevida do cateter em 1 ano
Jwo et al. (2010)^55^	Laparoscopia	37	10,8	NE	21,6	21	NE	NE	6,2	5,4	82
Kou et al. (2023)^56^	Laparoscopia	440	NE	5,9	NE	NE	NE	NE	NE	NE	NE
Iorga et al. (2024)^57^	Laparoscopia	126	4,3	NE	21,7	4,3	3,1	NE	30,4	NE	NE
Ìslam et al. (2023)^58^	Laparoscopia	24	25	NE	33,3	25	NE	NE	8,3	4,3	NE
Jwo et al. (2010)^55^	Laparotomia	40	17,5	NE	17,5	7,5	NE	NE	12,5	2,5	86
Chula et al. (2014)^27^	Laparotomia	42	NE	9,5	0	9,5	2,3	2,3	NE	0	76
Al-Hwiesh AK (2014)^59^	Laparotomia	40	7,5	12,5	2,5	0	0	0	17,5	2,5	87,5
Zhang et al. (2020)^60^	Laparotomia	114	0	0,9	NE	1,8	NE	NS	2,6	1,8	NE
Dogra et al. (2018)^61^	Laparotomia	105	3,8	0,9	0	7,6	NE	0	2,8	0,9	71,8
Aksu et al. (2007)^62^	Seldinger	108	9,2	5,5	NE	NE	NE	1,8	NE	5,5	92,4
Zou et al. (2021)^63^	Seldinger	94	22,5	15,5	9	2,2	NE	NE	NE	0	NE
Chula et al. (2023)^34^	Seldinger	73	6,8	0	0	3,9	1,3	0	3,9	2,6	95
Ìslam et al. (2023)^58^	Seldinger	36	19,4	NE	11,1	19,4	NE	NE	0	5,6	NE
Huang et al. (2022)^64^	Seldinger	34	0	2,9	2,9	17,6	0	0	0	2,9	NE
Dias et al. (2017)^65^	Seldinger	51	15,6	NE	7,8	NE	NE	0	17	3,8	NE
Chula et al. (2014)^27^	Trocarte	79	NE	12,6	0	6,3	2,5	2,5	NE	1,2	82
Lee et al. (2015)^66^	Trocarte	148	1,2	9,8	16,3	13,3	NE	NE	16,7	0	NE
Peng et al. (2019)^67^	Trocarte	84	NE	10,7	1,2	1,2	0	0	0	0	NE
Li et al. (2020)^68^	Trocarte	103	1	4,7	0	0	0	0	NE	3,8	92,2
Zhang et al. (2022)^69^	Trocarte	280	2,5	5,3	1	0	1,5	0	1	2,5	NE
Zhang et al. (2020)^70^	Trocarte	126	1,6	2,4	NE	1,6	NE	NE	0,8	1,6	NE

Abreviatura – NE: não especificado.

Em conclusão, a despeito dos avanços nas técnicas de implantação de cateteres para DP, persistem desafios relevantes. Os autores observam a necessidade de ensaios clínicos randomizados, bem delineados, que comparem de forma robusta as principais técnicas atualmente utilizadas. Paralelamente, é fundamental o uso sistemático de tecnologias e recursos de imagem, objetivando maior precisão técnica e redução de complicações. Além disso, o envolvimento ativo do nefrologista nesses procedimentos é essencial, tanto para otimizar os desfechos clínicos quanto para favorecer a expansão da DP como modalidade terapêutica.

## Data Availability

O presente artigo é uma revisão, baseada exclusivamente em dados secundários provenientes da literatura científica e em experiência profissional dos autores. Como não foram gerados dados primários nem compilados conjuntos de dados inéditos, não há arquivos adicionais a serem disponibilizados. Todas as informações analisadas podem ser consultadas nas referências citadas.
